# The relationship between working alliance with peer mentors and eating psychopathology in a digital 6‐week guided self‐help intervention for anorexia nervosa

**DOI:** 10.1002/eat.23559

**Published:** 2021-05-27

**Authors:** Gaia Albano, Valentina Cardi, Dennis M. Kivlighan, Suman Ambwani, Janet Treasure, Gianluca Lo Coco

**Affiliations:** ^1^ Department of Psychology, Educational Science and Human Movement University of Palermo Palermo Italy; ^2^ Department of Psychological Medicine Kings College London London UK; ^3^ Department of General Psychology University of Padova Padova Italy; ^4^ Department of Counseling, Higher Education and Special Education University of Maryland College Park Maryland USA; ^5^ Department of Psychology Dickinson College Carlisle Pennsylvania USA

**Keywords:** anorexia nervosa, cross‐lagged panel, eating psychopathology, guidance, mentors, online guided self‐help, peer mentors, working alliance

## Abstract

**Objective:**

The quality of working alliance (WA) is associated with treatment outcomes across several types of psychiatric disorders and psychological interventions. This study examined the role of WA with peer mentors (people with lived experience of illness) and student mentors (graduated psychology students) in a 6‐week, digital, guided self‐help (GSH) intervention for anorexia nervosa.

**Method:**

Ninety‐nine patients rated weekly, for 6 weeks: (a) eating psychopathology using the short version of the Eating Disorder Examination Questionnaire (EDE‐QS) and (b) WA with a student mentor (*n* = 14) or a peer mentor (*n* = 10). WA was assessed by asking patients the extent to which they felt comfortable working with their mentor and the extent to which they agreed with them on the goals for support. WA with mentors and the association with eating psychopathology change were measured on a session‐by‐session basis. The analysis involved a random intercepts cross‐lagged panel model.

**Results:**

WA with peer mentors was slightly higher than WA with students (ES = 0.3). Peer mentors' WA in the previous session was significantly associated with eating psychopathology ratings in the next session. No significant relationship was found between the previous session's EDE‐QS scores and peer mentor alliance in the following session. In the student mentor group, there were no session‐by‐session associations between WA and eating psychopathology. However, greater WA with the student mentor across sessions was associated with less eating psychopathology.

**Discussion:**

These findings suggest that clinical outcomes are in part associated with the characteristics of the mentor delivering guidance in an online GSH for eating disorders.

## 1 INTRODUCTION

Digital therapies have been largely tested in the field of eating disorders for the treatment of bulimia nervosa and binge‐eating disorders (Beintner, Jacobi, & Schmidt, 2014; Schlegl, Bürger, Schmidt, Herbst, & Voderholzer, 2015). Guided self‐help (GSH) is recommended for these conditions by the National Institute for Health and Care Excellence (NICE, 2017). GSH programs have the potential to supplement standard treatment through a variety of formats and modalities (face to face or online) usually involving mentors with past experiences of the illness or nonprofessionals. To date, GSH and digital interventions have been less studied in anorexia nervosa (AN), with experts in the field expressing concerns for patients’ safety (Wilson & Zandberg, 2012). Interestingly, recent meta‐analytic results demonstrate that the use of GSH in AN is associated with reduced drop‐out rates compared to Specialist Supportive Clinical Management or Treatment as usual (TAU) (OR [95% CI] = 0.63[0.41–0.95]), although there were only small effects for changes in body mass index (BMI), depression and anxiety (ES of 0.08, −0.07, and −0.03; Albano, Hodsoll, Kan, Lo Coco, & Cardi, 2019). In this study, we use data from the Self‐Help and Recovery guide for Eating Disorders (SHARED) trial (which was one of the studies included in Albano et al., 2019). The SHARED trial tested the use of a 6‐week digital GSH intervention to augment TAU for AN (i.e., RecoveryMANTRA; Cardi et al., 2015).

In the SHARED trial, RecoveryMANTRA guidance was provided by peer mentors (individuals with lived experience of eating disorders) or student mentors, with the overall goal of sharing information and encouraging behavior change. Patients receiving RecoveryMANTRA in addition to TAU reported a greater reduction in anxiety (small effect size) at the end of the intervention (6 weeks), but not at follow‐up (6 months), compared to TAU only (Cardi et al., 2019). Furthermore, patients receiving RecoveryMANTRA in addition to TAU reported greater confidence in their own ability to change (small effect size) and greater alliance with their mentor at the outpatient clinic (small‐to‐medium effect size) at 6 weeks, compared to the TAU only group (Cardi et al., 2019). Patients who dropped‐out early from the intervention were less satisfied with the online guidance received from the mentor delivering guidance at the end of the first week (Cardi et al., 2020). These results indicate that further research is needed to establish how effective mentoring strategies can be implemented in digital GSH for AN. In the current study, we examined how working alliance (WA) with the mentors delivering online guidance in the SHARED trial impacted on eating psychopathology.

The concept of WA includes the quality of the emotional bond established in the therapeutic dyad and also patient–therapist agreement about the goals of therapy (Zilcha‐Mano & Errázuriz, 2017). The relation of WA and treatment outcomes has been consistently evidenced across different psychotherapy treatments, and a meta‐analysis of 18 studies supported a predictive relation between alliance and outcomes (*d* = 0.57) in e‐mental health, with therapy delivered via Internet, e‐mail, or videoconferencing (Flückiger, Del Re, Wampold, & Horvath, 2018). In the eating disorder field, a recent meta‐analysis of 20 studies suggested that there is a bi‐directional relationship between symptom reduction and alliance in the early phase of treatment, especially for younger patients with AN (Graves et al., 2017). All the studies included in the meta‐analysis considered WA with a professional delivering therapy. Less clear is the impact of WA with less specialized individuals, including peer mentors, on clinical outcomes from GSH. Recently, there has been growing interest in understanding of how recovered individuals, also defined as peer mentors, can contribute to clinical change. There is some indication that peer mentorship can help patients feeling understood and improve clinical outcomes and treatment attendance (Beveridge et al., 2019; Perez, Van Diest, & Cutts, 2014; Ramjan, Fogarty, Nicholls, & Hay, 2018). A recent pilot randomized controlled trial (RCT) examining the feasibility and efficacy of peer mentorship for individuals with an eating disorder found a preference for, and higher engagement with peer mentors, compared to general social support mentorship (Ranzenhofer et al., 2020).

The current study is the first to examine the role of WA with both peer mentors and psychology student mentors over the course of digital online GSH for AN. We conducted a process analysis of data from the SHARED trial, with the overall goal of examining the association between WA with a student mentor or peer mentor and eating disorder outcomes, on a session‐by‐session basis. It is worth noting that the evidence on the association between WA and outcome in the treatment of EDs is still inconsistent across diagnoses, treatments, and time of assessment (Brauhardt, de Zwaan, & Hilbert, 2014; Graves et al., 2017), and there is a lack of research examining the role of WA for AN patient improvement across sessions. This is one of the first papers to explore day‐by‐day associations between WA and outcomes in eating disorders.

Zilcha‐Mano (2020) distinguished between trait‐like and state‐like measures of WA. Trait‐like WA captures patients' ability to form an alliance with the therapist or mentor and constitutes the between‐patient aspect of WA because it involves averaging WA across multiple occasions (Zilcha‐Mano, 2020). State‐like WA describes, instead, the within‐patient aspect of WA, which changes over time and has direct and specific associations with clinical outcomes (Zilcha‐Mano, 2020). The current study aimed to examine the contribution of trait‐ and state‐like WA in the treatment of patients with AN.

In this study, patients in the intervention arm (RecoveryMANTRA + TAU) had access to self‐help materials (workbook and short video‐clips) and received weekly online chat‐based guidance from a student mentor (i.e., a trained postgraduate student of psychology) or from a peer mentor (i.e., a recovered patient who suffered from an eating disorder). Patients were assigned to a mentor by the study team, based on mentors' availability, and patients did not choose nor were they randomized to a mentor type. Based on the literature on WA, we examined whether (a) higher alliance with the mentor/peer mentor in a session would predict lower eating psychopathology in the following session, and (b) the causal association between WA and eating psychopathology would be stronger for patients assigned to a peer mentor compared to those assigned to a postgraduate student mentor.

## 2 METHOD

This is a process data analysis from a multicenter randomized clinical trial for outpatients with a diagnosis of AN. The trial tested the efficacy of RecoveryMANTRA, a digital, 6‐week GSH intervention facilitated by student mentors or peer mentors. The trial design consisted of testing RecoveryMANTRA in addition to TAU versus TAU alone. Participants, who were randomly allocated to the intervention arm (RecoveryMANTRA + TAU condition), received self‐help materials and weekly guidance from mentors to supplement their TAU. The weekly guidance was delivered by mentors 1 hr/week, for 6 weeks, using online texting on a secure platform. The self‐help materials included a collection of short video clips and a self‐care workbook. The intervention materials were developed with the goal to increase confidence to change, internal motivation, connectedness to others, and hope. In the RecoveryMANTRA + TAU condition, patients were allocated to mentors on the basis of mentors' availability. Patients were blind to the type of mentoring they had been assigned to, and mentors were instructed not to reveal whether they had suffered from an eating disorder in the past. Participants allocated to the control group received the TAU provided by their participating centers (e.g., group‐based psychoeducation, individual psychotherapy, nutritional support, and medical monitoring). Full details of the study protocol are reported in Cardi et al. (2015), and the main outcome findings are reported in Cardi et al. (2019). The study involved human participants and was reviewed and approved by the Research Ethics Committee of London‐Brent.

### 2.1 Participants

Participants were involved in the trial if they met the following inclusion criteria: (a) they were aged 16 or over; (b) had a diagnosis of AN, or atypical or subclinical AN, based on the criteria of the Diagnostic and Statistical Manual of Mental Disorders, fifth edition (American Psychiatric Association, 2013); and a BMI of 18.5 kg/m^2^ or below; and (c) they had been referred, at the time of the recruitment, to 1 of the 22 UK outpatient eating disorder centers that participated in the trial.

Participants were considered ineligible if they had (a) insufficient knowledge of English; (b) severe mental or physical illness needing treatment (i.e., psychosis or diabetes mellitus); and/or (c) did not have access to the Internet. For the purposes of the present study, only patients in the treatment arm (*Recovery*MANTRA + TAU) were included into the analysis. These were 99 participants (97% were female), with a mean age of 26.60 (8.46) and 15.59 (2.83) years of education. At baseline assessment, 67/99 (74.4%) of patients had started outpatient treatment, their average BMI was 16.06 (1.44) and mean illness duration was 7.24 (8.81) years. Forty‐one participants (47.1%) were using psychiatric medications and 22 (25.9%) had had previous hospital admissions for their eating disorder.

### 2.2 Mentors/peer mentors

Twenty‐four mentors were engaged in the trial; 10 were individuals recovered from an eating disorder and 14 (2 male) were postgraduate psychology students who provided weekly online guidance through 1:1 synchronous chat sessions (i.e., online texting) for 6 weeks. All were aged above 19. All mentors attended training in motivational interviewing and received 1:1 weekly supervision by clinical psychologists and high‐qualified professionals in eating disorders for the whole duration of their involvement in the project. Online guidance was delivered once/week, for 6 weeks, through 1:1 written chat on the IESO Digital Health online platform (http://www.iesohealth.com). The goal of the online sessions was to guide participants through the use of the RecoveryMANTRA materials (workbook and short video‐clips). These materials were based on the cognitive interpersonal model of AN (Schmidt & Treasure, 2006; Treasure & Schmidt, 2013) and were aimed at providing psychoeducation and support goal setting in four areas: emotion regulation, social connection, cognitive flexibility, and healthy eating. In the treatment arm, patients were allocated to mentors on the basis of mentorship availability; that is, the maximum case load was three participants/mentor, from April, 2015 to December, 2016 (recruitment time).

### 2.3 Measures

Patients rated their eating psychopathology on a weekly basis, for 6 weeks, using the short version of the Eating Disorder Examination Questionnaire (EDE‐QS; Gideon et al., 2016). The original version of the EDE‐QS showed good internal consistency (Cronbach's *α* = .91) and temporal stability (ICC = 0.93; *p* = <.001), and was highly correlated with the original EDE‐Q (Fairburn & Beglin, 1994; Gideon et al., 2016). In the current study, Cronbach's alpha for the EDE‐QS total score was .81. Patients rated the perceived alliance with the mentor/peer mentor on a weekly basis, for 6 weeks, using a two‐item ultra‐brief visual analogue scale (VAS) ranging from 1 (never) to 7 (always). The items were “How often do you feel comfortable working with your mentor?” and “How often do you and your mentor agree on what needs to be done to improve your situation”? These items were adapted from the session rating scale used by Duncan et al. (2003) to measure bond and agreement on task. The two items were averaged to create a composite alliance score, and Cronbach's alpha values for the six time points ranged between .89 and .93.

### 2.4 Data analyses

We used the random intercepts cross‐lagged panel model (RI‐CLPM: Hamaker, Kuiper, & Grasman, 2015), implemented in Mplus. RI‐CLPM models between‐patient effects by including a random intercept for each of the variables (mentor/peer mentor alliance and EDE‐QS) (i.e., a factor with the six time loadings constrained to 1). This analysis is important with nested data sets with three levels (i.e., time [Level 1], patients [Level 2], and mentors/peer mentors [Level 3]). The random intercept in the CLPM removes between‐person variance (i.e., Level 2) such that the lagged relationships in the RI‐CLPM characterize within‐person change over time (i.e., Level 1; Hamaker et al., 2015). To address the nesting of patients within mentors/peer mentors, we followed the suggestion of McNeish, Stapleton, and Silverman (2017) and estimated cluster robust‐standard errors for Level 3 (mentor level). We used the grouping command in Mplus to estimate separate RI‐CLPM models for the student mentor and peer mentor groups.

Three fit indices were used to evaluate the fit of the model: the comparative fit index (CFI), root mean square error of approximation (RMSEA), and standardized root mean square residual (SRMR). According to the recommendations from Hu and Bentler (1999), criteria for acceptable fit have ranged from CFI ≥0.90 and SRMR and RMSEA ≤0.10, to more conservative criteria of CFI ≥0.95, SRMR ≤0.08, and RMSEA ≤0.06.

We estimated two models, one with the auto‐correlation and the cross‐lagged paths freely estimated and a second model with the auto‐correlation (e.g., Alliance1 → Alliance2 = Alliance2 → Alliance3 = Alliance3 → Alliance4…) and the cross‐lagged paths constrained to be equal across time periods (e.g., Alliance1 → EDE‐QS2 = Alliance2 → EDE‐QS3 = Alliance3 → EDE‐QS4…). The Santorra–Bentler scaled *χ* ^2^ different test (Muthén & Muthén, 2017) examined the difference between these nested models. Based on the parsimony principle, a nonsignificant *χ* ^2^ different test indicates that the constrained model (i.e., the auto correlation and cross‐lagged paths were set to be equal) is the preferred model.

The *χ* ^2^ different tests indicated the unconstrained model was not a significantly better fit to the data than the constrained model (*p* = .612). This constrained RI‐CLPM model had an adequate fit (*χ*2 = 165.462, *df* = 118, *p* = .003, CFI = 0.961, RMSEA [90% CI] = 0.091 [0.055, 0.122], SRMR = 0.109.

## 3 RESULTS

Twenty‐four mentors were recruited in the trial and each one supported a range of 1–13 (mean = 11.12; *SD* = 6.94) participants. Specifically, peer mentors (i.e., people with lived experience of the illness, *n* = 10) assisted 40 patients, while student mentors (*n* = 14) assisted 59 patients. The average rating of WA for peer mentors was slightly higher (*d* = 0.34) (mean = 5.56, *SD* = 1.12); than the average rating of WA for student mentors (mean = 5.13; *SD* = 1.32). The mean eating psychopathology scores (EDE‐Q) and BMI at baseline for participants assigned to peer mentors were 3.90 (*SD* = 1.22) and 16.14 (*SD* = 1.46), respectively; while they were 4.12 (*SD* = 1.04) and 16.02 (*SD* = 1.45) for patients assigned to student mentors. There was a small difference between groups for EDE‐Q ratings at baseline (*d* = − 0.19), but not for the BMI (*d* = 0.08).

### 3.1 Cross‐lagged panel model for student mentors

For the student mentor group, the mentor WA across all six sessions loaded significantly on the mentor WA random intercept (loadings ranged from 0.521 to 0.662, *p*s < 0.001). EDE‐QS ratings across the six sessions loaded significantly on the EDE‐QS random intercept (loadings ranged from 0.667 to 0.744, *p*s < 0.001). Between‐patient student mentor WA correlated significantly and negatively with between‐patient EDE‐QS, (−0.33, *t* = −2.26, *p* = .024). Therefore, when the trait‐like (i.e., across the six sessions) WA with the student mentor was stronger, patients reported less eating psychopathology.

**FIGURE 1:**
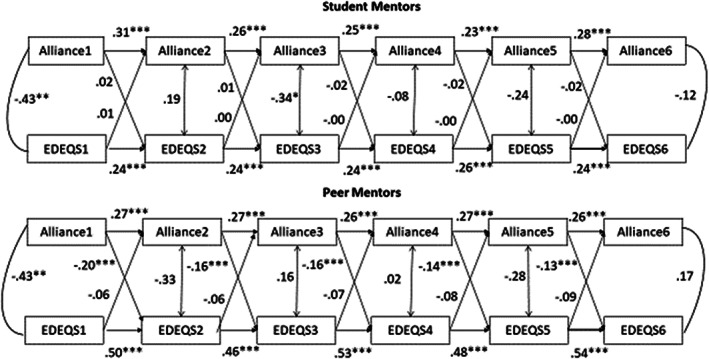
Random intercept cross‐lagged panel model with mentor alliance and eating psychopathology (EDE‐QS) at Sessions 1–6. Only the within‐person relationships are depicted in the figure. Estimates are standardized regression coefficients and covariances. Auto‐regressive and cross‐lagged paths were constrained to be time‐invariant. The cross‐lag relationships (lines between two points in time for different variables) depict the temporal ordering of variables. For example, if a patient's alliance increases between Week 2 and Week 3, compared to their own average WA change, do they decrease more in eating disorder symptoms from Weeks 3 and 4 than other weeks. The contemporaneous relationships (lines between two different variables at the same point in time) depict cross‐sectional relationships. For example, if a patient's alliance increases between Week 2 and Week 3, compared to their own average WA change, do they also decrease more in eating disorder symptoms from Weeks 2 and 3 than other weeks. *p < .05, **p < .01, ***p < .001

Figure 1 displays the significant standardized auto correlations, cross‐lagged paths, and within‐time period correlations. As seen in Figure 1, there were significant stability auto‐correlations for WA with students, for all measurements (e.g., between Times 1 and 2; 0.31, *t* = 5.82, *p* < .001). For student mentors, the cross‐lagged paths between early mentor alliance and later EDE‐QS ratings were all small and not significant (*p*s > 0.49). In addition, the cross‐lagged paths between early EDE‐QS and later mentor alliance were all small and not significant (*p*s > 0.50). Therefore, earlier mentor WA was not related to later eating psychopathology and earlier eating psychopathology was not related to later mentor WA at any point in time. With student mentors, state‐like changes in WA were not related to lower eating psychopathology. There were two types of cross‐sectional relationships between within‐mentor WA and eating psychopathology; at Session 1 (−0.43, *t* = −3.50, *p* < .001) and Session 3 (−0.34, *t* = −2.01, *p* = .044), WA was negatively and significantly correlated with EDE‐QS. Stronger alliance during these sessions was associated with less eating psychopathology at these sessions.

### 3.2 Cross‐lagged panel model for peer mentors

For the peer mentor group, peer mentor WA across all six sessions loaded significantly on the mentor WA random intercept (loadings ranged from 0.589 to 0.717, *p*s < .001). EDE‐QS ratings across the six sessions loaded significantly on the EDE‐QS random intercept (loadings ranged from 0.451 to 0.603, *p*s < .001). Between‐patient mentor WA correlated positively but not significantly with between‐patient EDE‐QS (0.35, *t* = 1.84, *p* = .067). Therefore, there was no relationship between trait‐like, peer mentor WA, and eating psychopathology.

Figure 1 displays the significant standardized auto correlations, cross‐lagged paths and within‐time period correlations. As seen in Figure 1, there was significant stability auto‐correlations) for peer mentor WA across all measurements, (e.g., between times 1 and 2 (0.27, *t* = 5.77, *p* < .001).

For peer mentors, the cross‐lagged paths between early mentor WA and later EDE‐QS ratings were all significant (e.g., between times 1 and 2 (−0.20, *t* = −3.98, *p* < .001). However, the cross‐lagged paths between early EDE‐QS and later peer mentor WA were all small and not significant (*p*s > 0.280). Therefore, if state‐like, peer mentor WA in a session was higher than usual, the eating psychopathology the following session was lower than usual for the patient. However, earlier eating psychopathology was not related to later WA with the peer mentor. With regard to cross‐sectional relationships, at Session 1 (−0.43, *t* = −3.05, *p* = .002) WA with the peer mentor was negatively and significantly correlated with EDE‐QS. Stronger alliance at Session 1 was associated with less eating psychopathology in that session.

## 4 DISCUSSION

This study analyzed the relationship between patients' perception of WA with their mentors/peer mentors and eating disorder psychopathology assessed on a session‐by‐session basis, over the course of a digital GSH intervention. The overall WA for the peer mentors was slightly higher (small effect) than WA for student mentors. We found a different temporal pattern of results for WA between student mentors or peer mentors. The cross‐lagged findings for the peer mentor group indicated that higher within‐patient WA in a session predicted lower eating psychopathology in the following session. Regarding the student mentor group, higher average patient's WA was associated with reduced eating psychopathology across all sessions. However, the cross‐lagged panel results for student mentors showed that earlier WA did not predict reduced eating symptoms the following session, and that earlier eating psychopathology was not related to later WA at any point in time. This is one of the first papers to explore the weekly associations between WA and clinical outcomes of AN patients undergoing GSH. Although the effectiveness of psychotherapeutic interventions for patients with AN has been documented (Zeeck et al., 2018), the outcomes and processes of change associated with the use of GSH need to be further established within this patient group (Traviss‐Turner, West, & Hill, 2017; Albano et al., 2019).

Previous research on the role of WA for patient improvement reported mixed findings, with some research supporting the influence of WA on patient symptom change ( Stiles‐Shields et al., 2013), and others suggesting the opposite (Brown, Mountford, & Waller, 2013). One explanation for these mixed findings is the failure to separate the effects of trait‐like (between‐patient) and state‐like (within‐patient) WA. The present findings indicate that the temporal patterns of influence between WA and the EDE‐Q outcome can depend on the type of mentorship received. In the current study, state‐like WA between patients and peer mentors with lived experience of the illness was associated with symptom change on a session‐by‐session basis. Although research on the role of peer mentorship in the treatment of AN is still scarce, it was recently suggested that peer mentorship can make patients feel understood and can provide sense of hope that improvement is possible, fostering positive affect and hopefulness (Ranzenhofer et al., 2020). Our findings add to this, suggesting that peer mentors can help patients getting better by enhancing WA at each session. It is possible that peer mentors acquired a relational expertise, which stems from their own lived experience, which can be useful to foster high patient engagement and a strong agreement on tasks and goals on a session‐by‐session basis. On the other hand, our findings suggest that student mentors are less effective to establish a strong WA with patients. These findings seem to support the importance of distinguishing between trait‐like and state‐like components of WA that characterize the individual patient (Zilcha‐Mano & Errázuriz, 2017). As described above, the trait‐like component of alliance refers to patient's ability to form satisfactory relationships with others, and it is examined as mean alliance level that the patient is able to form with the therapist (Zilcha‐Mano, 2020). In this regard, our findings indicate that this interpersonal ability is especially important when patients work with student mentors. On the other hand, the state‐like component of the alliance refers to changes in WA during treatment that can predict changes in clinical outcomes. In this regard, our findings suggest that changes in the state‐like component of WA are the result of in‐session work with the peer mentor, which in turn contributes to better outcomes. The distinction between WA with peer mentors or student mentors can pave the road to optimizing treatment efficacy and personalizing treatment (Zilcha‐Mano, 2020), by offering peer mentorship for those with lower state alliance particularly in the context of GSH. Previous research in psychological treatments has demonstrated that early WA between patient and therapist is associated with symptom change in young patients with AN (Graves et al., 2017). Our findings add to this literature demonstrating that this effect extends to adult patients also, regardless of how psychological support is delivered (online or face‐to‐face) (Berger, 2017; Sucala et al., 2012). Moreover, our findings showed an association between higher WA and lower eating symptoms at Session 1 both for the peer mentor and student mentor groups. This result indicates the relevance of early alliance in GSH for AN, and the importance of considering the extent to which patients feel comfortable and in agreement with their mentors/peer mentors at the beginning of treatment.

It is worth noting that when the WA with the student mentor was higher, across all six sessions, patients reported more improvement in eating psychopathology. Therefore, patients with stronger ability to build a positive WA with the student mentor are more likely to get better in GSH. This finding is in line with the characteristics of GSH, where patients are required to take an active role in driving the process of change (Falbe‐Hansen, Le Huray, Phull, Shakespeare, & Wheatley, 2009) and use their abilities and skills. However, WA at each session did not predict symptom improvement the following session. This might be due to a lack of specific therapeutic background for the mentor and repaired by additional preparatory training. Indeed, a recent review on self‐help in people with binge eating (Beintner et al., 2014) highlighted that receiving guidance from a specialized professional was associated with larger intervention effects on some clinical outcomes than nonspecialist guidance. Our cross‐lagged panel results suggest that patients receiving guidance from peer mentors are more responsive to strengthen a positive alliance every session and this in turn fosters an improvement of symptomatic behaviors the following week. However, given the limited evidence on the process of change in GSH for AN, further research is warranted to identify patient‐ and mentors‐related characteristics associated with clinical change (Albano et al., 2019; Traviss‐Turner et al., 2017).

### 4.1 Strengths and limitations

To the best of our knowledge, this is the first study to analyze patient and mentor/peer mentor contributions to WA in predicting clinical change in AN. We investigated the process of change over time, for 6 weeks, in the intervention arm of a large RCT of digital GSH in AN (Cardi et al., 2019). Some limitations must be considered. Firstly, we only measured self‐reported eating psychopathology to measure clinical change. Further research is needed to test whether WA is associated with different eating disorder outcome measures, such as BMI, depression and anxiety (Albano et al., 2019). In this current study, BMI was not included in the analyses because this information was not ascertained on a weekly basis. However, measures of global eating disorder psychopathology remain the most widely used to evaluate the efficacy of GSH in eating disorders (Traviss‐Turner et al., 2017). Secondly, nonstandardized VASs were used to assess WA. Although brief assessments have many practical benefits for repeated measurements compared to longer questionnaires, and our VAS demonstrated excellent internal consistency across the time points, a more comprehensive assessment of WA might better represent the breadth of the construct. Previous studies in the field have estimated that the shared variance among numerous measures of WA in eating disorders is less than 50% (Graves et al., 2017), and therefore further research is needed to establish which core measures of alliance would be most suitable to assess this construct over time. Further research is also needed to examine therapist ratings of alliance, given the scarce availability of these data in the eating disorders field (Brauhardt et al., 2014). Finally, further research with the aim of testing the differences in alliance between peer mentors and student mentors should adopt random assignment of patients to these conditions.

## 5 CONCLUSIONS

This study corroborates the importance of WA in GSH for AN. These findings suggest that WA with peer mentors is slightly higher than WA with student mentors. Most importantly, this study indicates that WA is associated with clinical change when established with peer mentors. Higher WA with peer mentors in a session predicted lower than usual eating psychopathology in the following session, over 6 weeks. Given the limitations of this study, further research is warranted to examine the specific peer mentor characteristics, which can help patients in the process of therapeutic change.

## CONFLICT OF INTEREST

The authors declare that the research was conducted in the absence of any commercial or financial relationships that could be construed as a potential conflict of interest.

## ETHICS STATEMENT

The study involved human participants and was reviewed and approved by the Research Ethics Committee of London‐Brent, project reference number: 14‐LO‐1347. The patients/participants provided their written informed consent to participate in this study.

## Data Availability

The data sets generated for this study are available on request to the corresponding author.
